# Molecular Insights into Oxidative-Stress-Mediated Cardiomyopathy and Potential Therapeutic Strategies

**DOI:** 10.3390/biom15050670

**Published:** 2025-05-06

**Authors:** Zhenyu Xiong, Yuanpeng Liao, Zhaoshan Zhang, Zhengdong Wan, Sijia Liang, Jiawei Guo

**Affiliations:** 1 Department of Vascular and Endovascular Surgery, The First Affiliated Hospital of Yangtze University, Jingzhou 434000, China; 2 Department of Pharmacology, School of Medicine, Yangtze University, Jingzhou 434023, China; 3 Department of Pharmacology, Cardiac and Cerebral Vascular Research Center, Zhongshan School of Medicine, Sun Yat-sen University, Guangzhou 510080, China

**Keywords:** cardiomyopathies, oxidative stress, reactive oxygen species, antioxidants

## Abstract

Cardiomyopathies comprise a heterogeneous group of cardiac disorders characterized by structural and functional abnormalities in the absence of significant coronary artery disease, hypertension, valvular disease, or congenital defects. Major subtypes include hypertrophic, dilated, arrhythmogenic, and stress-induced cardiomyopathies. Oxidative stress (OS), resulting from an imbalance between reactive oxygen species (ROS) production and antioxidant defenses, has emerged as a key contributor to the pathogenesis of these conditions. ROS-mediated injury drives inflammation, protease activation, mitochondrial dysfunction, and cardiomyocyte damage, thereby promoting cardiac remodeling and functional decline. Although numerous studies implicate OS in cardiomyopathy progression, the precise molecular mechanisms remain incompletely defined. This review provides an updated synthesis of current findings on OS-related signaling pathways across cardiomyopathy subtypes, emphasizing emerging therapeutic targets within redox-regulatory networks. A deeper understanding of these mechanisms may guide the development of targeted antioxidant strategies to improve clinical outcomes in affected patients.

## 1. Introduction

According to a scientific statement published by the American Heart Association in 2006, cardiomyopathies are primarily classified into two major categories: primary and secondary cardiomyopathies. Primary cardiomyopathies are further subdivided into genetic, mixed, and acquired types [1]. In 2008, the European Society of Cardiology refined this classification by excluding congenital heart disease, hypertension, valvular heart disease, and coronary artery disease from the definition of cardiomyopathies [2]. The occurrence of cardiomyopathies is influenced by multiple factors, including infections, metabolic disorders, endocrine diseases, ischemia, and allergic reactions [3]. Recent studies suggest that mitochondria play a critical role in the onset of doxorubicin (Dox)-induced cardiomyopathy [4], septic cardiomyopathy [5], and diabetic cardiomyopathy [6]. Mitochondria are also the primary site of ROS production, particularly in cardiac muscle cells [7,8]. OS arises from the dysregulation of ROS [9]. Excessive ROS production causes oxidative damage to molecules, contributes to aging, and is implicated in the onset of various diseases, including cancer, neurodegenerative disorders, and cardiovascular diseases. Elevated ROS levels can shift the cellular redox balance towards oxidation, leading to cellular dysfunction and, in severe cases, apoptosis. Fortunately, the body possesses mechanisms to counteract OS through antioxidants. These antioxidants, which are either synthesized endogenously or obtained exogenously, detect ROS and mitigate oxidative damage in affected cells [10,11]. Aging is an important but underappreciated contributor to the pathogenesis of cardiomyopathies, particularly those driven by OS. With advancing age, mitochondrial efficiency declines, antioxidant defenses such as Nrf2 activity are attenuated, and ROS clearance capacity is reduced [12]. These age-related impairments in redox homeostasis render cardiomyocytes increasingly susceptible to oxidative injury. Diastolic dysfunction is especially prevalent in the elderly and often coexists with metabolic and inflammatory comorbidities, further emphasizing the role of aging in OS-mediated cardiac remodeling [13]. OS provides a novel perspective on the progression of cardiomyopathies [14]. This review highlights the role of OS in various cardiomyopathies and explores antioxidative strategies as potential targets for preventing and treating these conditions.

## 2. Sources and Biomarkers of OS in Cardiomyopathies

### 2.1. ROS-Generating Systems Contributing to Cardiomyopathy

#### 2.1.1. Mechanisms of ROS Generation in Cardiomyocytes

**Figure 1:**
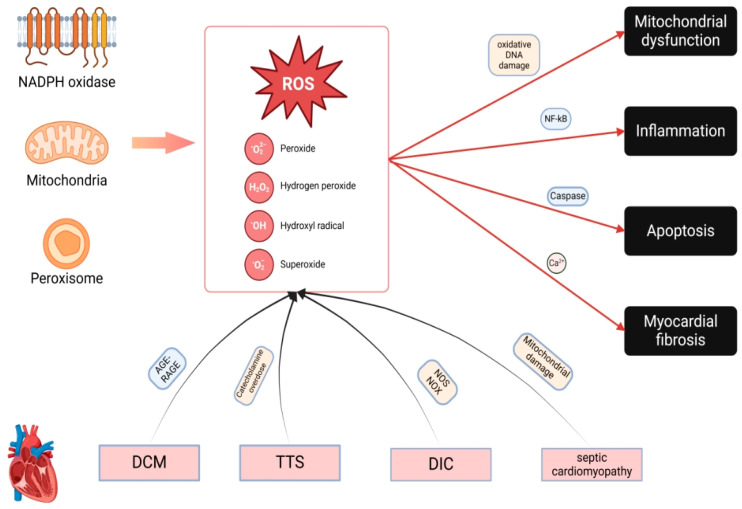
Pathogenic Mechanisms of ROS in Cardiomyopathies. ROS, including superoxide (O_2_⁻), hydrogen peroxide (H_2_O_2_), and hydroxyl radicals (OH·), originate primarily from mitochondria and NADPH oxidases (NOX), particularly NOX2. Excessive ROS production damages proteins, lipids, and DNA, impairing mitochondrial function and activating NF-κB signaling, which promotes inflammation, increases mitochondrial membrane permeability, and triggers apoptosis via cytochrome c release and caspase activation. In diabetic cardiomyopathy, hyperglycemia-induced ROS generation via the AGE-RAGE pathway exacerbates OS. Stress-induced cardiomyopathy is driven by excessive catecholamine release, while doxorubicin-induced cardiomyopathy involves ROS overproduction through NOS and NOX dysfunction. In septic cardiomyopathy, impaired mitochondrial electron transport amplifies ROS generation. This persistent oxidative damage creates a vicious cycle of mitochondrial dysfunction, accelerating cardiomyopathy progression.

ROS encompass non-radical compounds such as hydrogen peroxide, hypochlorous acid, and ozone, as well as oxygen radicals like superoxide anions, hydroxyl radicals, and peroxyl radicals [15]. Mitochondria are the primary producers of oxidants within most cell types (Figure 1) [16]. Nicotinamide adenine dinucleotide phosphate (NADPH) oxidases, particularly the NOX enzyme family (notably, NOX2 and NOX4), are critical contributors to OS in cardiovascular diseases. Intracellular ROS production is further amplified by the activity of various enzymes, including xanthine oxidase (XO), nitric oxide synthases (NOSs), cyclo-oxygenases, cytochrome P450 enzymes, and lipoxygenases. Additionally, organelles such as peroxisomes and the endoplasmic reticulum contribute to ROS production [17]. Both ROS and reactive nitrogen species (RNS) primarily impact cellular components like proteins, lipids, and DNA. The production of molecular oxygen as ROS is an inherent aspect of aerobic life. Baseline ROS levels are essential for numerous cellular processes, including signal transduction, microbial defense, gene expression, and the regulation of cell growth and programmed cell death. Although redox reactions are vital for maintaining physiological functions, their dysregulation can exacerbate pathological conditions, including aging. To mitigate ROS-induced damage, the body employs a range of defense mechanisms, including enzymatic antioxidants (e.g., superoxide dismutase, catalases, peroxiredoxins, and glutathione peroxidases) and non-enzymatic antioxidants (e.g., tocopherols/vitamin E, β-carotene, ascorbic acid, glutathione, and nicotinamide) [18]. Recent advancements in research methodologies have enabled the precise chemical exploration of redox signaling pathways, highlighting their importance in biological systems. Redox signaling is now recognized as a regulatory mechanism comparable to phosphorylation–dephosphorylation, acetylation–deacetylation, and methylation–demethylation, which govern genomic and epigenomic processes [19].

#### 2.1.2. Loss of Antioxidant Defense

In addition to increased ROS production, a decline in endogenous antioxidant defenses significantly contributes to OS in cardiomyopathies. Enzymatic antioxidants such as superoxide dismutases (SOD1/SOD2), catalase, and glutathione peroxidase (GPX) are downregulated in pathological cardiac states, often due to epigenetic or transcriptional repression [20]. Notably, reduced Nrf2 activity—an essential transcription factor for antioxidant gene regulation—has been observed in aged and diabetic myocardium. This loss in redox balance enhances the susceptibility of cardiomyocytes to ROS-induced injury and underlies the progression of myocardial fibrosis and dysfunction [21].

In chronic metabolic diseases such as diabetes and with advancing age, the antioxidant defense system becomes progressively impaired not only due to a reduced expression of antioxidant enzymes, but also via the dysregulation of upstream regulators. The epigenetic silencing of Nrf2 target genes through promoter hypermethylation and histone deacetylation has been observed in diabetic myocardium [22]. Moreover, the overactivation of Keap1—a cytosolic inhibitor of Nrf2—enhances Nrf2 degradation and blocks its nuclear translocation, thereby limiting the transcription of critical antioxidant genes such as SOD1, GPX4, and HO-1 [23,24]. These changes further tilt the redox balance toward a pro-oxidative state and exacerbate cardiac vulnerability to stress.

#### 2.1.3. Synergy of ROS Sources

While mitochondria represent the primary quantitative source of ROS in cardiomyocytes, NADPH oxidases (particularly NOX2 and NOX4) serve as critical signaling hubs in redox biology. Cross-talk between ROS sources is increasingly recognized; for example, NOX-derived ROS can disrupt mitochondrial membrane potential and trigger further mitochondrial ROS generation, a process referred to as “ROS-induced ROS release”. This synergistic amplification creates a feed-forward loop of oxidative injury, potentiating cellular dysfunction [25]. Moreover, NOX activation has been shown to promote mitochondrial fission and cytochrome c release, further linking the cytosolic and mitochondrial OS pathways [26]. Importantly, the spatial and temporal dynamics of ROS generation from different sources may determine the specificity of redox signaling and cell fate.

### 2.2. Biomarkers of OS in Cardiomyopathy

OS plays a central role in the pathogenesis of cardiomyopathies by inducing structural and functional impairments in cardiomyocytes [27]. The excessive production of ROS leads to oxidative modifications of lipids, proteins, and nucleic acids, contributing to cellular dysfunction and disease progression [28]. The identification and quantification of OS biomarkers provide valuable insights into the redox status of the myocardium and hold potential for diagnostic and prognostic applications in cardiomyopathies.

#### 2.2.1. Lipid Peroxidation Markers

Lipid peroxidation, a hallmark of oxidative damage, occurs when ROS react with polyunsaturated fatty acids in cell membranes, leading to the formation of reactive aldehydes such as malondialdehyde (MDA) and 4-hydroxy-2-nonenal (4-HNE) [29]. MDA, a widely used biomarker of OS, can form adducts with proteins and nucleic acids, exacerbating cellular dysfunction and apoptosis [30]. Increased MDA levels have been reported in patients with diabetic cardiomyopathy and doxorubicin-induced cardiotoxicity, correlating with disease severity [31]. Similarly, 4-HNE, a highly reactive electrophile, modulates intracellular signaling pathways and induces mitochondrial dysfunction, further contributing to cardiomyocyte injury [32].

#### 2.2.2. Protein Oxidation Markers

Oxidative modifications of proteins alter their structure and function, impairing enzymatic activity and disrupting intracellular signaling. 3-nitrotyrosine (3-NT), a biomarker of nitrosative stress, is formed through the peroxynitrite-mediated nitration of tyrosine residues in proteins [33]. Elevated 3-NT levels have been detected in heart failure and hypertrophic cardiomyopathy, reflecting excessive peroxynitrite production and impaired nitric oxide homeostasis [34]. Additionally, carbonylated proteins, generated through the direct ROS-mediated oxidation of amino acid side chains, serve as stable indicators of protein oxidative damage [35]. Increased protein carbonylation is associated with mitochondrial dysfunction and apoptosis in cardiomyopathies, underscoring its potential as a biomarker of disease progression.

#### 2.2.3. DNA Damage Markers

OS-induced DNA damage contributes to genomic instability and impaired cellular repair mechanisms in cardiomyocytes [36]. 8-hydroxy-2′-deoxyguanosine (8-OHDG) is a widely recognized marker of oxidative DNA damage, formed when ROS attack guanine residues in DNA [37]. Elevated 8-OHdG levels have been observed in both experimental models and patients with cardiomyopathies, correlating with increased myocardial fibrosis and apoptosis [38]. Persistent oxidative DNA damage can activate the p53 signaling pathway, leading to cardiomyocyte senescence and heart failure [39].

#### 2.2.4. Emerging Redox Biomarkers and Their Clinical Significance

Beyond conventional OS markers, emerging redox biomarkers provide novel insights into redox homeostasis and cardiomyopathy progression [40]. The circulating glutathione (GSH/GSSG) ratio, a key indicator of intracellular redox balance, has been proposed as a prognostic marker in heart failure [41]. Additionally, mitochondrial ROS indicators, such as superoxide production and mitochondrial DNA damage, have gained attention for their role in early-stage cardiomyopathy detection [42]. Advances in metabolomics and redox proteomics have facilitated the identification of novel OS-related biomarkers, paving the way for precision medicine approaches in the management of cardiomyopathies [43].

Importantly, a major challenge in redox biomarker development is achieving tissue specificity. Most circulating OS markers reflect systemic redox imbalance and may not directly correlate with local myocardial injury. Emerging paradigms propose cardiac-specific oxidative modifications as potential biomarkers—for instance, oxidative adducts on cardiac myosin binding protein-C (cMyBP-C) have been implicated in diastolic heart failure [44]. Conversely, it is also plausible that cardiomyopathies may result from oxidative insults originating at distant sites, such as adipose tissue, endothelium, or immune cells, which subsequently propagate to the myocardium via circulating mediators. These contrasting mechanisms warrant further investigation [45].

A recent study highlighted the diagnostic potential of circulating protein oxidation markers in cardiovascular diseases [46]. Specifically, the quantification of oxidized serum albumin and advanced oxidation protein products (AOPPs) was shown to correlate with cardiac dysfunction severity and systemic oxidative load. These markers, being non-invasive and easily measurable, may complement traditional lipid and DNA oxidation indicators, particularly in conditions such as diabetic HFpEF where chronic OS is diffuse and systemic. Further validation is needed to establish their cardiac specificity and prognostic value.

In summary, lipid peroxidation, protein oxidation, and DNA damage markers serve as crucial indicators of OS in cardiomyopathies. The integration of conventional and emerging biomarkers may improve early diagnosis, risk stratification, and therapeutic monitoring, ultimately enhancing patient outcomes. Future research should focus on validating these biomarkers in large clinical cohorts and exploring their potential as therapeutic targets in OS-related cardiomyopathies.

### 2.3. Pathophysiological Effects of ROS on Cardiomyocyte Function

Mechanistically, OS impairs cardiac function through multiple interrelated pathways. Oxidative damage to mitochondrial components compromises ATP synthesis, reducing energy availability for myocardial contraction and relaxation [47]. ROS-induced post-translational modifications of ryanodine receptors (RyR2) alter the calcium release from the sarcoplasmic reticulum, contributing to calcium mishandling and contractile dysfunction [48]. Furthermore, oxidative modifications of contractile proteins, including tropomyosin and actin, disrupt sarcomere integrity and impair myocardial performance [49]. These mechanisms may explain the limited efficacy of general antioxidant therapies, which may not adequately reach specific subcellular compartments or sufficiently target dominant ROS sources.

## 3. OS in Specific Cardiomyopathy Subtypes

Given the heterogeneous nature of cardiomyopathies, the contribution of OS varies by subtype, severity, and comorbid metabolic conditions. In this section, we examine the role of OS in key cardiomyopathy phenotypes—including diabetic, stress-induced, chemotherapeutic, infectious, and genetic forms—highlighting the distinct ROS sources, downstream signaling, and therapeutic implications in each (Table 1).

### 3.1. OS in DCM: Molecular Mechanisms and Pathophysiological Insights

#### 3.1.1. OS and DCM

**Figure 2:**
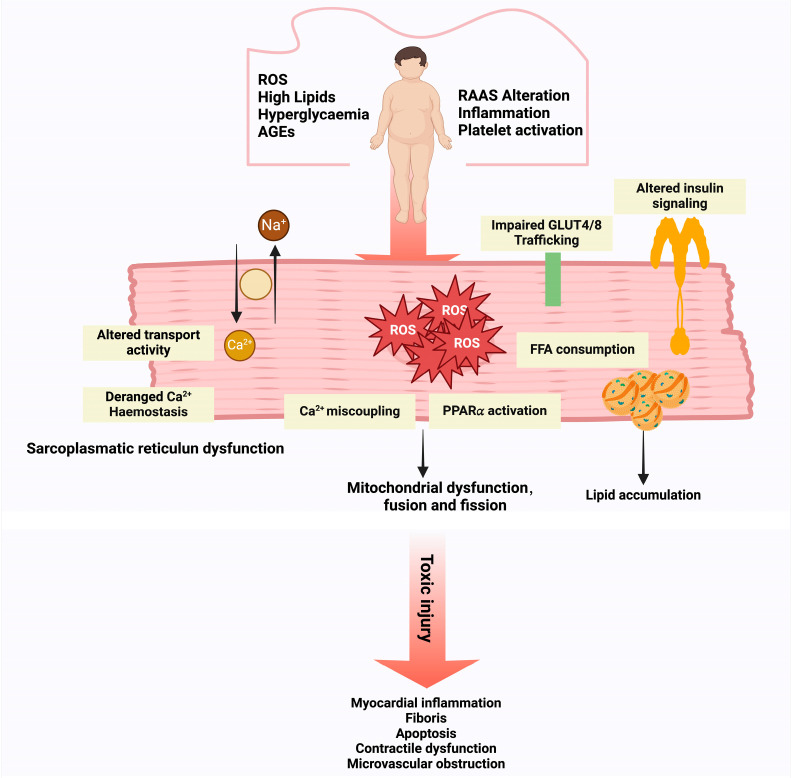
Pathophysiological Mechanisms of DCM. Hyperglycemia, ROS, dyslipidemia, advanced glycation end products, and alterations in the RAAS are primary contributors to the development of DCM. These factors disrupt calcium ion transport, leading to calcium homeostasis imbalance and mitochondrial dysfunction, both of which contribute to cardiotoxicity. Concurrently, impaired insulin signaling and dysregulated free fatty acid metabolism result in lipid accumulation, further exacerbating the pathological changes. Together, these mechanisms promote myocardial inflammation, fibrosis, apoptosis, contractile dysfunction, and microvascular obstruction, culminating in the clinical manifestation of DCM.

DCM is a pathological condition caused by diabetes mellitus that can lead to heart failure [50]. Hyperglycemia, insulin resistance, and hyperinsulinemia are independent risk factors for DCM [51]. The progression of cardiomyopathy in diabetes is driven by pathophysiological mechanisms, including systemic metabolic imbalances, the aberrant activation of the renin–angiotensin–aldosterone system (RAAS), the dysfunction of subcellular components, OS, inflammatory responses, and impaired immune regulation [52]. These abnormalities contribute to interstitial fibrosis, myocardial stiffness, diastolic dysfunction, impaired systolic function, and the eventual development of clinical heart failure syndrome (Figure 2) [53]. In humans, ROS are primarily generated in the heart through mitochondria, NOX, XO, uncoupled nitric oxide synthase, microsomal P-450 enzymes, and arachidonic acid metabolism pathways [54]. Excessive ROS production induces mitochondrial dysfunction, decreases fatty acid oxidation, and leads to lipid accumulation, fibrosis, diastolic dysfunction, and heart failure in diabetic patients [55]. The interplay between OS, endoplasmic reticulum stress, and disrupted calcium handling accelerates cardiomyocyte death through apoptosis, necrosis, and autophagy [56]. Increased ROS levels enhance mitochondrial outer membrane permeability, triggering apoptosis [57]. Excessive calcium uptake leads to calcium overload, which activates mitochondrial permeability transition pores, inducing apoptosis [58]. Furthermore, impaired autophagy, including defects in the fusion of autophagosomes and lysosomes in cardiomyocytes, is closely associated with systolic and diastolic dysfunction in DCM [59]. Endothelial dysfunction in coronary arteries, the dysregulation of exosomes, the activation of pro-inflammatory immune cells, and the inappropriate activation of the RAAS also play critical roles in the pathogenesis of DCM [60].

#### 3.1.2. Pathophysiologic Effects of Nox on DCM

The Nox family consists of seven isoforms, NOX1, NOX2, NOX3, NOX4, NOX5, Doux1, and Doux2, with NOX4 being the most extensively studied [61]. NOX4 plays a critical role in ROS production in the heart and is present in various heart cell types, including cardiomyocytes, endothelial cells, fibroblasts, and vascular smooth muscle cells [62,63]. The overactivation of NOX4 is strongly associated with cardiac injury in DCM [64]. Studies have shown that NOX4 expression is significantly increased in myocardial tissues of both type 1 diabetes mellitus and type 2 diabetes [64,65,66,67,68,69,70,71]. The overproduction of ROS due to NOX4 overactivity is linked to declines in heart function and structural damage [64,72]. In vitro studies have indicated that a significant reduction in NOX4 activity can decrease NADPH oxidase activity in cardiomyocytes [73], preventing the increase in fibrotic markers such as fibronectin, collagen, α-SMA, and β-MHC, and mitigating myocardial hypertrophy caused by hyperglycemia. These findings highlight the potential of NOX4 antisense oligonucleotides in controlling ROS and reducing early structural and functional damage in diabetic heart tissue [70]. In myocardial fibrotic remodeling, matrix metalloproteinases are crucial and are continuously induced and activated in DCM, serving as a key indicator of myocardial fibrosis [74]. In vitro experiments have demonstrated that high glucose levels elevate NOX4 expression, phosphorylated ERK1/2 levels, and both mitochondrial and total cellular ROS production in rat cardiac fibroblasts. Silencing NOX4 using siRNA significantly reduces the phosphorylated ERK1/2 and MMP-2 expression levels [75], suggesting that the NOX4-ROS-ERK signaling pathway plays an essential role in the fibrotic process of DCM. Targeting NOX4 may, therefore, be an effective strategy for preventing and treating DCM. Additionally, research has highlighted the relationship between angiotensin II (Ang II) and NOX4 in diabetic heart tissues and cells. Ang II receptor blockers have been shown to reduce the overexpression of angiotensin type 1 receptor, NOX4, and p22phox in the heart tissues of diabetic rats induced by streptozotocin (STZ), lowering OS and the expression of the profibrotic factor transforming growth factor-β (TGF-β), thereby improving heart function [76]. However, further investigation is needed to fully elucidate these interactions. Studies in diabetic hamsters have shown significant increases in chymase, 8-hydroxy-2′-deoxyguanosine (8-OHdG), and NOX4 expression, alongside elevated myocardial Ang II levels and fibrosis markers. The inhibition of chymase restores Ang II levels in the myocardium of STZ-induced diabetic hamsters, reversing NOX4-driven OS and myocardial fibrosis [77]. This suggests that NOX4 plays a role in Ang II-induced myocardial fibrosis in DCM, and targeting Ang II or NOX4 could serve as a promising therapeutic approach for DCM. Moreover, TGF-β, a potent pro-fibrotic cytokine in cardiac cells, is closely associated with the development of cardiac fibrosis and heart failure. In animal and cellular models of DCM, NOX4 upregulation is often correlated with increased TGF-β expression [64,69,76,78,79,80]. Ang II and TGF-β are essential for the development of myocardial hypertrophy and fibrosis, with Ang II inducing TGF-β expression in cardiomyocytes and fibroblasts [81,82,83]. Furthermore, Nox oxidases are crucial intracellular signaling molecules required for the Ang II-triggered elevation of TGF-β in cardiomyocytes [84]. These findings indicate that Ang II, TGF-β, and Nox oxidases function within a signaling network that regulates cardiac remodeling processes. Research on how advanced glycation end products interact with NOX4 in DCM remains limited. However, studies indicate that, in myocardial tissue from diabetic rats induced by isoproterenol, both the AGE receptor (RAGE) and NOX4 expression are significantly elevated, with both being regulated by peroxisome proliferator-activated receptor gamma (PPARγ) [85].

#### 3.1.3. OS and Diastolic Dysfunction in DCM

While DCM is traditionally characterized by left ventricular dilation and reduced systolic function, clinical and epidemiological data suggest that diastolic dysfunction is far more prevalent in individuals with diabetes. This phenotype, often categorized within the spectrum of heart failure with preserved ejection fraction (HFpEF), is strongly associated with chronic low-grade OS [86]. ROS can impair diastolic function through several mechanisms: the oxidation of sarcomeric proteins such as titin increases myocardial stiffness; the reduced nitric oxide bioavailability impairs endothelial-mediated relaxation; and the oxidative modifications of calcium-handling proteins—including SERCA2a and phospholamban—disrupt intracellular calcium reuptake during diastole [87,88]. Furthermore, ROS promotes fibroblast activation and collagen deposition, contributing to increased myocardial wall rigidity. It is plausible that differing magnitudes and spatial patterns of OS contribute to the phenotypic divergence between systolic and diastolic heart failure [89]. Specifically, modest but sustained OS may preferentially drive diastolic dysfunction, whereas more severe or mitochondrial-localized ROS could trigger systolic impairment and cardiomyocyte death.

#### 3.1.4. Immune-Cell-Derived OS in DCM

In addition to cardiomyocytes, immune cells—particularly circulating and tissue-resident white blood cells (WBCs)—play a significant role in initiating and amplifying OS in DCM. Neutrophils and monocyte-derived macrophages express high levels of NOX2 and myeloperoxidase (MPO), both of which are potent sources of ROS [90,91]. In the setting of metabolic stress, such as hyperglycemia or insulin resistance, these immune cells infiltrate the myocardium and contribute to a pro-oxidative and pro-fibrotic microenvironment [92]. Recent studies in murine models of HFpEF have demonstrated that the depletion of WBCs or the inhibition of myeloid NOX2 reduces the myocardial ROS burden and improves diastolic function [93,94]. These findings suggest that WBC-derived ROS may act as an upstream trigger of endothelial dysfunction, fibroblast activation, and the oxidative modification of extracellular matrix components. Importantly, the contribution of immune-cell-mediated OS may exceed that of cardiomyocyte-derived ROS in certain phenotypes, such as HFpEF associated with diabetes or obesity [95].

### 3.2. OS and TTS

TTS, also known as stress cardiomyopathy or broken heart syndrome, was first identified in Japan in 1990 and is recognized as a significant form of acute, reversible myocardial injury, characterized by the transient regional systolic dysfunction of the left ventricle (LV) [96,97,98]. TTS is marked by acute, temporary LV systolic dysfunction lasting less than 21 days, often triggered by emotional or physical stress, with the maximum severity typically occurring within 1 to 5 days of onset [99]. The diagnostic criteria most commonly used for TTS are those from the Heart Failure Association of the European Society of Cardiology [100], although international diagnostic criteria for TTS have recently been proposed [101,102]. Patients with TTS may exhibit diverse clinical manifestations, with the syndrome frequently triggered by significant emotional stress or severe physical illness, leading to the activation of the sympathetic nervous system [103]. A substantial interplay exists between psychological stress and cardiovascular diseases, primarily driven by the increased release of stress hormones, the generation of OS, and the activation of inflammatory pathways, ultimately leading to vascular and endothelial dysfunction [104]. These pathophysiological mechanisms are thought to play a critical role in the characteristic systolic narrowing of the LV and apical ballooning in TTS [101]. These contractile abnormalities are usually transient, resolving, in most cases, within a few months following the event [102]. Notably, the early recovery of LV dysfunction in TTS patients, even without coronary intervention, suggests a transient stunned myocardium, without the typical complications of acute coronary syndrome, such as myocardial scarring. This phenomenon may be linked to increased OS. In TTS, ischemia-induced ROS generation or elevated catecholamine release is thought to contribute to myocardial damage [105]. The direct exposure of cardiomyocytes to ROS has been shown to result in the loss of contractility, progressive diastolic dysfunction, the inhibition of metabolic function, and the depletion of high-energy phosphates [106,107]. Compared to acute MI patients, studies have demonstrated that TTS patients exhibit elevated levels of norepinephrine and 8-OHdG in the coronary sinus, which is not observed in the aortic root or peripheral vasculature. On the first day following TTS, 8-OHdG levels positively correlate with the wall motion score index, an indicator of LV dysfunction, and increase as the wall motion score index rises [108]. Gene expression changes in TTS patients provide further evidence of ROS involvement in myocardial stunning. Nef et al. observed that, during the acute phase of TTS, there is an increase in the expression of genes activated by nuclear factor erythroid 2-related factor 2 (Nrf2), which is activated by ROS [109,110]. Nrf2, a transcription factor responsive to OS, regulates gene transcription through thiol-based control by Keap1 [111]. By activating gene transcription via antioxidant response elements, Nrf2 regulates the expression of several antioxidant genes, including ascorbic acid, superoxide dismutase (SOD), catalase, and glutathione peroxidase 1 [112]. These findings suggest that ROS activation in the acute phase of TTS leads to an increase in antioxidant defense mechanisms [113]. The Nrf2-mediated ROS detoxification pathway is considered the most powerful internal antioxidant signaling mechanism, effectively preventing OS-induced endothelial dysfunction [114]. Recent studies have indicated a notable increase in central macrophage infiltration in TTS patients [115]. Macrophages, which contain phagocytic NOX-2, produce superoxide, a key regulator of OS in heart and blood vessel tissues [116]. Ueyama et al. investigated the role of OS in TTS and observed the significant upregulation of heme oxygenase-1 (HO-1) in a stress-induced rat model. HO-1, a well-established marker of OS, is commonly found in atherosclerosis [117]. Research has shown that HO-1 offers antioxidative and anti-inflammatory benefits by producing heme breakdown products, such as carbon monoxide and biliverdin/bilirubin, and by increasing ferritin expression [118]. While macrophages play a crucial role in OS induction, they also express β- and α-adrenergic receptors. Studies have demonstrated that pretreatment with β- and α-adrenergic receptor antagonists significantly reduces HO-1 expression and alters gene expression, exerting cardioprotective effects. Using a β-adrenergic receptor agonist, isoproterenol, Mao et al. established a TTS model and found that OS and apoptosis levels in the myocardial tissue were significantly elevated. Other studies confirmed that mitochondria are a major source of superoxide [119]. Zhang et al. showed that isoproterenol-induced LV dysfunction was associated with decreased myocardial and circulating hydrogen sulfide levels, the upregulation of NADPH oxidase subunits NOX-4 and NOX-2, and increased circulating hydrogen peroxide concentrations. Additionally, reduced levels of lipid peroxidation markers (MDA) and decreased reduced glutathione (GSH) indicated OS. Notably, NOX-4, not confined to the cytosol, enhances ROS production in mitochondria upon upregulation, promoting apoptosis [120]. The administration of sodium thiocyanate improved isoproterenol-induced cardiac dysfunction and reversed OS, restoring the normal expression of NADPH oxidase subunits. These findings suggest that H_2_S exerts cardioprotective effects by reducing ROS production, mitigating OS mediated by NADPH oxidase inhibition [121]. Furthermore, studies by Willis et al. demonstrated a significant increase in mitochondrial-derived ROS production in rats treated with isoproterenol [122].

### 3.3. OS and DIC

Cardiovascular adverse events are among the most common and significant side effects of cancer therapy [123]. Studies indicate that cardiotoxicity in patients undergoing cancer treatment can lead to asymptomatic LV dysfunction, cardiomyopathy, and progressive heart failure, accounting for approximately 60% of cancer-related cardiovascular mortality [124]. Although Dox is a highly effective anticancer agent, its clinical use is limited by cumulative and persistent cardiotoxic effects [125]. The primary mechanisms of DIC include the excessive generation of ROS and Top2β toxicity, which together cause double-strand breaks in DNA and mitochondrial damage [126,127,128,129]. Currently, the only cardioprotective agent approved by the U.S. Food and Drug Administration to mitigate Dox-induced cardiotoxicity is dexrazoxane, which reduces OS by chelating free iron [125]. While dexrazoxane has demonstrated efficacy in reducing cardiotoxicity caused by anthracyclines, its use has been restricted in pediatric patients due to an increased risk of secondary malignancies [130,131]. Therefore, it is essential that we investigate the biological mechanisms underlying DIC to develop safer and more effective drugs that can overcome the limitations of conventional chemotherapy. This study primarily explores the role of Nox signaling in DIC and its potential therapeutic effects.

Nox is the cytochrome component of phagocyte NAD(P)H oxidase, which is essential for producing ROS [132]. NOX2 and NOX4 facilitate the one-electron reduction in the Dox quinone structure to form a semiquinone intermediate, which subsequently reacts with oxygen to generate O_2_·−, H_2_O_2_, and ·OH, leading to damage to the heart muscle [133]. For catalytic activity, Nox must bind with multiple subunits, as it lacks intrinsic catalytic activity. NOX2 activation requires five components, p22phox, p67phox, p40phox, p47phox, and the GTPase Rac, with p22phox being central [134]. In contrast, NOX4 activation necessitates the participation of p22phox and the polymerase Poldip2 [135,136]. Dox can trigger the NOX signaling pathway, increasing the levels of NOX2 and NOX4, which enhances ROS production, thereby exacerbating OS and further stimulating the MAPK pathway, leading to cell apoptosis [137]. Moreover, Dox stimulates Drp1 by upregulating NOX1 and NOX4, initiating mitochondrial fission, which activates the NLRP3 inflammasome in cardiomyocytes, resulting in pyroptosis [138]. Studies have shown that inhibiting NOX2 and NOX4 expression effectively reduces excessive ROS production, alleviating DIC [139,140]. Rac, as a key regulatory factor, plays an essential role in NOX2 activation. The activation of Rac triggers a feedback loop that self-activates NOX2, leading to an oxidative burst and a significant increase in ROS production [141]. The knockout of Rac inhibits NOX activation, reduces ROS generation, and alleviates DIC. Additionally, the application of the specific Rac inhibitor NSC23766 has been shown to provide cardioprotection in DIC mice [142]. p67phox also contributes to NOX2 activation. Research by Zhang et al. demonstrated that Irisin, by inhibiting the expression of p67phox, can reduce NOX activation and activity, thereby mitigating OS [143]. This plays a crucial role in regulating NOX expression. Valsartan, by inhibiting the Ang II receptor, downregulates NOX2 and NOX4 expression, thus reducing the incidence of DIC [137].

### 3.4. OS and Septic Cardiomyopathy

Sepsis is characterized by life-threatening organ dysfunction resulting from an abnormal host response to infection. Septic shock, which occurs in the context of severe abnormalities in circulatory, metabolic, and cellular function, is typically marked by the need for vasopressor support and persistent hyperlactatemia in the absence of hypovolemia [144]. Sepsis-induced cardiac dysfunction, widely referred to as septic cardiomyopathy, has long been a focus of research [145]. Septic cardiomyopathy involves LV dilation, typically with normal or lowered filling pressures and a reduced ejection fraction. This condition is reversible and generally begins to improve within 7 to 10 days after onset [146]. Potential contributors to myocardial depression in sepsis include hypoxia, acidosis, hypotension, decreased blood volume, metabolic disturbances, coagulopathy, and increased levels of ROS and RNS [146]. Most of the oxygen within cells is absorbed by mitochondria and utilized for ATP production, suggesting that these organelles play a pivotal role in the pathophysiology of sepsis-induced organ dysfunction [147]. A significant association exists between OS and human sepsis, correlating strongly with disease severity and mortality rates [148]. Sepsis-induced myocardial dysfunction is closely linked to the overproduction of ROS and RNS [149,150,151]. The imbalance in the oxidative state leads to the excessive production of ROS, NO, and their toxic derivatives, such as peroxynitrite, which are major contributors to myocardial injury [152]. OS occurs when the production of ROS and RNS exceeds the antioxidant defense system’s capacity, leading to mitochondrial dysfunction [12]. Cardiomyocytes contain mitochondria in their cytoplasm, which primarily generate energy via oxidative phosphorylation [153]. Sepsis disrupts the respiratory chain’s capacity, causing an imbalance in energy metabolism. During oxidative phosphorylation, a small amount of O_2_− is generated and subsequently converted into H_2_O_2_ by manganese superoxide dismutase, a crucial intracellular antioxidant. Mitochondria, the key organelles for energy regulation in heart muscle cells, are essential in initiating and sustaining energy imbalances during sepsis, leading to heart dysfunction [154]. The generation of ROS induces alterations in the mitochondrial ultrastructure and function, some of which result in irreversible mitochondrial failure, ultimately contributing to multi-organ dysfunction [155,156]. NOX, consisting of membrane-bound catalytic subunits and several cytosolic regulatory subunits, shows heightened activity during septic responses, especially when stimulated by lipopolysaccharides. Notably, studies have demonstrated a close association between the severity of mitochondrial dysfunction and the clinical severity of sepsis [157,158,159]. Excessive ROS and RNS production triggers lipid peroxidation, protein oxidation, nitration reactions, and DNA strand breaks. Consequently, an OS imbalance can compromise cellular membrane integrity, affecting enzyme function and gene expression. Furthermore, excessive ROS and RNS generation in mitochondria has been shown to inhibit oxidative phosphorylation, resulting in reduced ATP production [160]. Poly (ADP-ribose) polymerase (PARP) can be activated by oxidative and nitrosative stress, leading to a decrease in the LV systolic function index [161]. The overproduction of ROS causes lipid oxidation, which affects membrane integrity. In a time-based study using a rat model of pneumonia-induced sepsis, it was found that sepsis led to progressively severe oxidative damage in cardiac mitochondria. The mitochondrial outer membrane damage and cytochrome c release confirmed this finding. Moreover, the reduction in the activities of antioxidant enzymes, such as SOD and GPX, resulted in a marked increase in oxidative reactions affecting lipids and proteins in mitochondria [162]. Oxidative damage to lipids and proteins is a critical factor contributing to myocardial structural alterations and the clinical manifestations of septic cardiomyopathy. These changes appear to precede phenotypic alterations associated with septic cardiomyopathy. Studies have demonstrated that membrane damage, as an early event in severe sepsis induced by cecal ligation and puncture (CLP) in mice, results in increased plasma membrane permeability [163]. Additionally, mitochondrial damage induced by sepsis may contribute to vacuolization in cardiomyocytes, suggesting apoptosis in these cells [164]. Further studies have confirmed that the loss of mitochondrial structural integrity is a key feature of the septic response, characterized by the disorganization of the mitochondrial cristae and the swelling of the mitochondrial matrix [161,165]. The role of mitochondria in inflammation regulation is primarily mediated through the activation of NF-κB, a key mediator of apoptosis. The activation of NF-κB is closely associated with its translocation from the cytoplasm to the nucleus. During sepsis, cytoplasmic levels of NF-κB decrease, while nuclear levels increase, indicating its activation [162,164]. MAVS and DOC-4, proteins found in the mitochondrial matrix, are involved in controlling NF-κB activation, while alterations in mitochondrial calcium levels can influence cytokine production in heart muscle cells [166,167,168]. The inflammatory response in infectious myocarditis progressively intensifies, preceding alterations in myocardial mitochondria. This observation suggests that sepsis-induced myocardial mitochondrial damage occurs as a cascade, encompassing cytochrome c release, outer membrane disruption, increased lipid and protein oxidation, and diminished mitochondrial ROS defense. These events collectively culminate in progressive myocardial inflammation and advanced cardiac dysfunction [162].

### 3.5. Other OS-Associated Cardiac Disorders

Beyond the primary cardiomyopathy subtypes discussed earlier, OS has also been implicated in several other cardiac pathologies, including HCM, MI, and cardiac arrhythmias. In HCM, both inherited and sporadic forms exhibit increased oxidative burden linked to mitochondrial dysfunction and impaired antioxidant defenses [169]. Mutations in sarcomeric proteins such as MYH7 and MYBPC3 have been associated with enhanced ROS production, which contributes to cardiomyocyte hypertrophy and fibrotic remodeling [170]. Similarly, during MI, OS is a central mediator of ischemia-reperfusion injury [171]. The abrupt reintroduction of oxygen leads to excessive ROS generation, predominantly from dysfunctional mitochondria and xanthine oxidase activity, which exacerbates myocardial necrosis and promotes post-infarction remodeling [172].

Furthermore, OS plays a critical role in the genesis of cardiac arrhythmias, which account for nearly half of all heart-failure-related deaths [173]. ROS can alter electrophysiological stability by modifying key ion channels, including the ryanodine receptor (RyR2), Na⁺ channels, and L-type Ca^2+^ channels [174]. These modifications increase the intracellular Ca^2+^ leak, delay repolarization, and promote the triggered activity. Mitochondria-derived ROS has also been linked to action potential prolongation and impaired conduction, fostering arrhythmogenic substrates [153]. Collectively, these findings highlight the multifaceted contribution of OS to diverse forms of cardiac pathology and underscore the need for redox-targeted interventions beyond traditional heart failure paradigms.

## 4. Targeting OS: Therapeutic Strategies and Future Directions

Therapeutic approaches targeting OS can be broadly categorized based on their mechanism of action: the inhibition of ROS production, the enhancement in endogenous antioxidant capacity, metal chelation, and the protection of vulnerable organelles such as mitochondria. This section systematically summarizes pharmacological and natural agents within these categories, focusing on their mechanisms, targets, and relevance to specific cardiomyopathy subtypes.

OS is a fundamental driver of cardiomyopathies, contributing to mitochondrial dysfunction, inflammation, fibrosis, and cardiomyocyte death [175]. While ROS play essential roles in physiological signaling, their excessive production and inadequate detoxification disrupt cardiac homeostasis. The multifaceted nature of OS in cardiomyopathies necessitates targeted therapeutic strategies, ranging from ferroptosis inhibition and antioxidant supplementation to iron chelation and gene therapy [176]. Despite promising preclinical findings, translating these therapies into clinical practice remains challenging due to the intricate interplay between redox homeostasis, metabolic pathways, and immune responses [177]. This section explores state-of-the-art therapeutic strategies aimed at mitigating OS in cardiomyopathies, emphasizing molecular mechanisms, pharmacological interventions, and future directions in redox medicine (Table 2).

### 4.1. Ferroptosis and OS in Cardiomyopathies

Ferroptosis, a distinct form of regulated cell death characterized by iron-dependent lipid peroxidation, has been increasingly recognized as a critical contributor to cardiomyocyte injury in various cardiomyopathies [195]. Unlike apoptosis or necrosis, ferroptosis is primarily driven by the accumulation of lipid peroxides, which disrupt cellular membranes and induce mitochondrial dysfunction [196]. In cardiomyopathies, excessive ROS generation, often exacerbated by mitochondrial dysfunction and dysregulated iron homeostasis, acts as a catalyst for ferroptotic cell death [197]. The Fenton reaction, in which free iron reacts with hydrogen peroxide (H_2_O_2_) to generate hydroxyl radicals (OH·), plays a pivotal role in amplifying oxidative damage [198].

In DIC, doxorubicin enhances iron accumulation in mitochondria, leading to excessive ROS production and lipid peroxidation, ultimately triggering ferroptosis [199]. Similarly, in DCM, hyperglycemia-induced OS exacerbates iron overload, further promoting ferroptotic pathways [200]. Septic cardiomyopathy also exhibits ferroptosis-related damage, as systemic inflammation increases ROS production while impairing antioxidant defense mechanisms [201]. Given the significance of ferroptosis in cardiac pathology, ferroptosis inhibitors such as ferrostatin-1, liproxstatin-1, and zileuton (a 5-lipoxygenase inhibitor) have been explored for their cardioprotective effects [202]. These agents work by blocking lipid peroxidation and preserving mitochondrial integrity. Additionally, the modulation of the glutathione peroxidase 4 (GPX4) pathway, a key regulator of ferroptosis, has emerged as a promising therapeutic target [203]. Enhancing GPX4 activity through selenium supplementation or pharmacological upregulation could provide novel therapeutic avenues for OS-induced cardiomyopathies [204].

### 4.2. Antioxidant-Based Therapeutic Strategies

The human body possesses intricate antioxidant defense mechanisms that counteract oxidative damage. However, in cardiomyopathies, excessive ROS production overwhelms these defenses, necessitating external therapeutic interventions [205]. Antioxidant-based therapies primarily focus on enhancing endogenous antioxidant systems or supplementing exogenous antioxidants to neutralize ROS and restore redox homeostasis [206].

Endogenous antioxidant defense mechanisms are primarily governed by the Nrf2 (nuclear factor erythroid 2-related factor 2) signaling pathway, which regulates the expression of antioxidant enzymes such as SOD, catalase (CAT), and GPX [207]. The pharmacological activation of Nrf2 using agents such as bardoxolone methyl, sulforaphane, and dimethyl fumarate has shown promise in reducing OS-related cardiac damage [208]. In addition, SOD mimetics like tempol and MnTBAP have been developed to enhance the dismutation of superoxide anions, mitigating mitochondrial oxidative damage [209].

Exogenous antioxidants, including polyphenols, vitamins, and synthetic compounds, have been widely investigated for their cardioprotective effects [210]. Polyphenolic compounds such as resveratrol, quercetin, and curcumin exhibit potent ROS-scavenging properties and modulate signaling pathways involved in inflammation and fibrosis [211]. Vitamins C and E, known for their antioxidant activity, have demonstrated some efficacy in reducing oxidative damage; however, clinical trials have produced mixed results, highlighting the complexity of redox regulation [212]. Mitochondria-targeted antioxidants, such as MitoQ and SkQ1, have gained attention for their ability to selectively neutralize ROS within mitochondria, thus preventing oxidative damage at its primary source [213]. Additionally, N-acetylcysteine (NAC), a precursor of glutathione, has been explored for its capacity to replenish intracellular antioxidant stores and mitigate cardiomyocyte oxidative injury [214].

Magnesium, an essential cofactor for numerous mitochondrial enzymes, has demonstrated cardioprotective effects in OS-related models of DCM [215]. By stabilizing the mitochondrial membrane potential and limiting calcium overload, magnesium reduces ROS generation and mitigates oxidative injury [216]. Preclinical studies indicate that magnesium supplementation improves diastolic function and reduces myocardial fibrosis [217]. These findings suggest that magnesium may serve as an adjunctive antioxidant strategy in metabolic cardiomyopathies.

Gene therapy approaches targeting the redox balance have also been explored as potential long-term interventions for OS-related cardiac dysfunction [218]. Strategies involving the overexpression of antioxidant enzymes (e.g., SOD2 and GPX4) or the suppression of pro-oxidant genes (e.g., NOX2 and p66Shc) using CRISPR/Cas9 or RNA interference have shown preclinical promise in reducing oxidative damage and improving cardiac function [219].

### 4.3. Iron Chelation and Redox Homeostasis

Iron plays a dual role in cellular physiology: while essential for numerous biochemical processes, excess iron contributes to OS via the Fenton reaction [220]. Dysregulated iron homeostasis has been implicated in several cardiomyopathies, particularly in conditions such as hereditary hemochromatosis, doxorubicin-induced cardiotoxicity, and DCM, where excess iron amplifies ROS production [221].

Iron chelation therapy has emerged as a promising strategy for mitigating iron-driven oxidative damage in cardiomyopathies [222]. Deferoxamine, a clinically approved iron chelator, has demonstrated cardioprotective effects by reducing labile iron pools and preventing ROS-induced mitochondrial damage [223]. However, its poor bioavailability and short half-life have prompted the development of alternative chelators such as deferasirox and deferiprone, which offer improved pharmacokinetics and tissue penetration [224].

Beyond traditional chelation therapy, recent studies have explored the potential of novel iron regulators, such as hepcidin mimetics and ferroportin inhibitors, to modulate iron homeostasis and reduce oxidative damage in cardiomyopathies [225]. Additionally, natural iron chelators, including hinokitiol and quercetin, have shown promise in preclinical models by stabilizing the redox balance and preventing iron-mediated cardiotoxicity [226].

### 4.4. The Role and Mechanism of Ferroptosis in AD

Mitigating OS and enhancing antioxidant defenses have been shown to effectively alleviate cardiomyopathy. Melatonin can attenuate the progression of DCM by preserving mitochondrial quality, with the melatonin-membrane-receptor-mediated SIRT6-AMPK-PGC-1α-AKT axis playing a pivotal role in this process (Table 3) [227]. Additionally, melatonin reduces DCM by increasing autophagy in cardiomyocytes, and its cardioprotective effects depend on the VEGF-B/GRP78/PERK signaling pathway [228]. The activation of the Sirt1/Nrf2 pathway is a potential mechanism through which melatonin mitigates OS, pyroptosis, and apoptosis in DIC [229]. Furthermore, studies have shown that melatonin can synergize with agents such as antibiotics, resveratrol, and antioxidants to reduce inflammation and improve cardiac function, offering therapeutic benefits for septic cardiomyopathy [230]. Combination therapy with alpha-lipoic acid (ALA), gliclazide, and ramipril has been shown to prevent the onset of DCM by inhibiting the TGF-β1/Smad pathway [231]. In lipopolysaccharide-induced endotoxemia rat models, a vitamin C pretreatment significantly reduced MDA levels, restored SOD activity, decreased inflammatory biomarkers, and alleviated myocardial cell injury [232]. Quercetin and vitamin E mitigate DCM by inhibiting the mitochondrial permeability transition (mPT) pore opening and regulating mitochondria-mediated apoptosis [233]. The administration of the antioxidant N-acetylcysteine (NAC) for five weeks in STZ-induced type 1 diabetes mellitus rat and mouse models normalized OS levels and prevented the progression of DCM [234]. NAC exhibits both antioxidant and anti-inflammatory properties, mitigating Dox-induced cardiac and renal damage, and showing therapeutic potential for periapical periodontitis in patients with nephropathy and cardiomyopathy [235]. The oral administration of ferulic acid significantly improves various metabolic disturbances in models of DCM [236]. Treatment with the SOD mimetic MnTBAP prevents superoxide-induced cardiac pathological changes in PPARγ knockout mice [237].

### 4.5. Iron-Chelator-Related Targets and Drugs

In cardiomyocytes, the mitochondrial overexpression of GPX4 or Fe^2+^ chelation has been shown to prevent Dox-induced ferroptosis, indicating that Dox triggers ferroptosis within mitochondria. The co-inhibition of ferroptosis and apoptosis using ferrostatin-1 (Fer-1) and zVAD-FMK completely prevents Dox-induced cardiomyocyte death [245]. Fer-1, in combination with dexamethasone (DXZ), also inhibited ferroptosis in mice with septic cardiac injury [255]. Targeting the FTO/BACH1 axis and employing ferroptosis inhibitors effectively mitigated septic cardiomyopathy [246]. In vitro studies have shown that lipopolysaccharides induce myocardial cell contraction and abnormal lipid peroxidation, which can be reversed by the mitochondrial autophagy inducer ursolic acid, the inhibition of ACSL4, and ferroptosis modulation [256]. Ferroptosis also plays a crucial role in the pathogenesis of DCM; sulforaphane prevents ferroptosis and related mechanisms through AMPK-mediated NRF2 activation, effectively preventing DCM [257]. The mitochondrial-targeted antioxidant MitoTEMPO significantly rescues DIC, supporting mitochondrial oxidative damage as the primary mechanism of ferroptosis-induced cardiac injury [249].

### 4.6. Novel Drug Targets and Emerging Therapies

Empagliflozin significantly improves myocardial OS damage and myocardial fibrosis in diabetic mice by inhibiting OS and converting the TGF-β/Smad pathway while activating the Nrf2/ARE signaling pathway to suppress neurofibrosis [250]. Resveratrol serves as an upstream regulator of multiple molecular signaling pathways, such as Sirt1/3, AMPK, Akt, and MAPK pathways. Modifications in these pathways exert cardioprotective effects, including antioxidant stress, anti-apoptosis, and autophagy promotion, thereby alleviating cardiac hypertrophy in diabetic patients and improving cardiac function [258]. Some flavonoid compounds, such as catechins, isoliquiritigenin, galangin, and luteolin, exhibit anti-inflammatory and antioxidant properties, potentially mediated through the Nrf2 signaling pathway, offering potential for the treatment of DCM [252,253,259,260]. The non-β-blocker carvedilol significantly reduces LV contractility by inhibiting RyR2, while also maintaining the stroke volume through α1-adrenergic receptor inhibition in vivo [254].

Recent advances in redox medicine have identified new therapeutic targets that extend beyond conventional antioxidants and iron chelation [261]. One of the most promising strategies involves targeting ROS-producing enzymes, particularly NOX, which are major sources of OS in cardiomyopathies [262]. NOX2 and NOX4 isoforms have been implicated in myocardial remodeling and fibrosis, making them attractive therapeutic targets [263]. NOX inhibitors such as apocynin, GKT137831, and VAS2870 have demonstrated efficacy in preclinical models by reducing oxidative damage and preserving cardiac function [264].

Mitochondria-targeted therapies have also gained momentum, as mitochondrial dysfunction is a primary driver of OS in cardiomyopathies [265]. Compounds such as SS31, a mitochondrial-protective peptide, and MitoTEMPO, a mitochondria-specific superoxide scavenger, have been investigated for their potential to restore mitochondrial integrity and improve bioenergetic function [266]. These agents help maintain the mitochondrial membrane potential, reduce ROS-induced damage, and enhance ATP production, thereby mitigating oxidative cardiotoxicity [267].

Personalized medicine approaches have begun integrating redox biomarkers to tailor OS-targeted therapies for individual patients [268]. Biomarkers such as 8-hydroxy-2′-deoxyguanosine (8-OHdG), 4-hydroxynonenal (4-HNE), and ox-LDL provide insights into the severity of oxidative damage and the efficacy of therapeutic interventions [268]. Future strategies may leverage high-throughput screening technologies and artificial intelligence to identify novel redox modulators and optimize patient-specific treatment regimens [269].

As research continues to elucidate the complex interplay between OS and cardiomyopathy, the development of multi-targeted therapeutic strategies that integrate ferroptosis inhibition, antioxidant therapy, iron regulation, and mitochondrial protection will be crucial. Future investigations should focus on refining the specificity of redox-targeted interventions, improving drug bioavailability, and overcoming translational challenges to ensure clinical efficacy. By harnessing advances in molecular biology, pharmacology, and precision medicine, novel therapies targeting OS hold great promise for improving outcomes in patients with cardiomyopathy.

### 4.7. Mechanisms of Antioxidant Treatment Failure

Despite promising results in preclinical studies, clinical trials targeting OS have yielded mixed or disappointing outcomes in patients with cardiomyopathies. Several explanations have been proposed. First, many antioxidants lack tissue specificity and fail to reach intracellular compartments where ROS are most abundantly generated, such as mitochondria or the sarcoplasmic reticulum [270]. Second, the heterogeneity of ROS species and sources—ranging from NOX-derived superoxide to mitochondrial hydrogen peroxide—necessitates precise molecular targeting that general antioxidants cannot achieve [271]. Third, ROS are not solely deleterious; they also participate in physiological signaling. Non-selective ROS scavenging may thus disrupt redox-sensitive homeostatic pathways [272]. These limitations highlight the need for redox precision medicine, involving subcellular targeting, selective inhibition, and combination strategies tailored to the disease context.

## 5. Outlook and Future Perspective

Current antioxidant drugs have shown some effectiveness in alleviating OS but still face issues such as poor efficacy, significant side effects, and difficulty in long-term use. Research targeting specific OS signaling pathways, such as NOX, mitochondria, or specific antioxidant enzymes (e.g., SOD and catalase), could enhance treatment selectivity and reduce off-target effects on non-target tissues. Existing antioxidants primarily mitigate OS by neutralizing free radicals, but OS is not only mediated by free radicals; it may also occur through other mechanisms, such as intracellular redox states and the regulation of specific signaling pathways. Signal pathways like Nrf2, NF-κB, and SIRT1 may become key targets for future therapies. Mitochondria are a major source of OS, and mitochondrial damage plays a key role in various types of cardiomyopathy. Therefore, studying mitochondria-specific antioxidants, such as mitochondrial-targeted SOD mimetics or antioxidant enzymes, could directly reduce ROS production and cellular damage. Increasing evidence suggests that inflammation and OS interact to drive the progression of cardiomyopathy. Future research could explore strategies combining antioxidant and anti-inflammatory treatments. For instance, combining antioxidants with inhibitors of inflammatory factors such as IL-6 and TNF-α may effectively alleviate both OS and inflammation in cardiomyopathy.

## 6. Conclusions

In summary, OS plays a fundamental role in the pathogenesis of cardiomyopathies by promoting mitochondrial dysfunction, inflammation, fibrosis, and cardiomyocyte death. Significant advances have clarified key molecular players, including NOX isoforms, ferroptosis mediators, and antioxidant pathways such as Nrf2. However, major gaps remain: the precise spatial and temporal contributions of ROS sources, tissue-specific biomarkers, and optimal therapeutic targets are still under active investigation. Future research should focus on developing mitochondria-targeted antioxidants, validating cardiac-specific redox biomarkers, and integrating anti-inflammatory and metabolic strategies. Such multifaceted approaches hold promise for translating the redox biology into effective therapies for cardiomyopathy.

## 7. Limitations

This review is based on a narrative methodology and does not employ a formal systematic review framework. As such, there is a possibility of selection or interpretation bias, despite our efforts to provide a balanced and representative overview of the current literature. Additionally, due to the rapidly evolving nature of OS research, some recent studies may not have been captured at the time of writing.

## Figures and Tables

**Table 1:** Comparative overview of OS mechanisms in different types of cardiomyopathies.

Cardiomyopathy Type	Key Mechanisms of Damage	Representative Biomarkers	Therapeutic Targets/Strategies
**Diabetic Cardiomyopathy (DCM)**	Fibrosis (TGF-β/ERK), impaired Ca^2+^ handling, diastolic dysfunction	4-HNE, 8-OHdG, MDA	Nrf2 activation, NOX4 inhibition, Mg^2+^
**Takotsubo Syndrome (TTS)**	Transient contractile dysfunction, mitochondrial stress	HO-1, ROS, SOD	β-blockers, antioxidant enzymes
**Doxorubicin-Induced Cardiomyopathy (DIC)**	Ferroptosis, mitochondrial fission, pyroptosis	ROS, iron, 3-NT	Ferroptosis inhibitors, iron chelators
**Septic Cardiomyopathy**	Inflammation, mitochondrial dysfunction, PARP activation	8-OHdG, nitric oxide, MDA	NOX inhibition, PARP inhibitors
**Hypertrophic Cardiomyopathy (HCM)**	Sarcomeric dysfunction, hypertrophy, fibrosis	Protein carbonyls, GSH/GSSG	Mito-targeted antioxidants, MYBPC3-related
**Myocardial Infarction (MI)**	Ischemia-reperfusion injury, inflammation, necrosis	MDA, LDH, MPO	Preconditioning, mitochondria protection

**Table 2:** Targeting OS in cardiomyopathies: key therapeutic strategies and drug candidates.

Therapeutic Strategy	Mechanism of Action	Representative Drugs	References
**Ferroptosis Inhibition**	Prevents lipid peroxidation, protects mitochondria	Ferrostatin-1	[178]
		Liproxstatin-1	[179]
		Zileuton	[180]
**Antioxidant Therapy**	Enhances endogenous antioxidant system, scavenges ROS	Bardoxolone methyl	[181]
		Sulforaphane	[182]
		MitoQ	[183]
		NAC	[184]
**Iron Chelation Therapy**	Reduces free iron, decreases ROS production	Deferoxamine	[185]
		Deferasirox	[186]
		Quercetin	[187]
**NOX Inhibition**	Inhibits NADPH oxidase, reduces oxidative damage	Apocynin	[188]
		GKT137831	[189]
**Mitochondrial Protection**	Maintains mitochondrial function, reduces OS	SS31	[190]
		MitoTEMPO	[191]
**Precision Therapy**	Optimizes interventions based on biomarkers	4-HNE	[192]
		8-OHDG	[193]
		ox-LDL	[194]

**Table 3:** Primary mechanisms and treatment targets in cardiomyopathy linked to OS.

Antioxidant	Mechanism	Target	References
** Vitamin C **	Reduction in inflammatory biomarkers and attenuation of cardiomyocyte damage.	OS	[232]
**Vitamin E**	Regulation of mitochondria-mediated apoptosis.	Cytochrome c	[233]
**Zinc**	Reduced cardiac morphological damage and fibrosis.	Metallothionein	[238]
**SFN**	Reduces cardiac OS, hypertrophy, and fibrosis.	Nrf2	[235,239]
** NAC **	Reduced myocardial OS, reduced cardiac hypertrophy and fibrosis.	OS	[240]
**mito-TEMPO**	Reduces cardiac mitochondrial ROS production and OS; reduces cardiomyocyte apoptosis and cardiac hypertrophy.	Superoxide	[241]
**Broccoli sprouts**	Reduced plasma IL-6 and CRP levels.	Nrf2	[242]
**chrysin**	Reduces myocardial OS, inflammation and apoptosis.	Nox4	[85]
**GKT137831**	Mild inhibition of Hi Glu/ThmG-induced ROS.	Nox4	[243]
**Melatonin**	Inhibits OS and apoptosis, and enhances autophagy.	Nrf2	[227,228,229,230]
**ALA**	Blocking apoptosis, oxidation, and inflammatory responses.	NF-κB	[244]
**Quercetin**	Regulation of mitochondria-mediated apoptosis.	Cytochrome c	[233]
**MnTBAP**	Prevention of superoxide-induced cardiac pathology in PPARγ knockout mice.	PPARγ	[237]
**Fer-1**	Inhibition of ferroptosis and apoptosis.	BACH1	[245,246]
**DXZ**	Cardioprotective, anti-inflammatory, and antioxidant.	free iron	[247,248]
**MitoTEMPO**	Prevents mitochondrial oxidative damage.	Mitochondria	[249]
**Empagliflozin**	Significantly ameliorated myocardial OS injury and myocardial fibrosis in diabetic mice.	Nrf2	[250]
**DHY**	Suppressed OS, inflammation and necrosis.	SIRT3	[251]
**ISL**	Attenuates cardiac hypertrophy, fibrosis, and apoptosis.	Nrf2	[252]
**Luteolin**	Modulation of the inflammatory response.	Nrf2	[253]
**Carvedilol**	Arrhythmia suppression.	RyR2	[254]
** SFN ** : Sulforaphane **Fer-1**: Ferrostatin-1 **DXZ**: Dexamethasone **DHY**: Dihydromyricetin **ISL**: Isoliquiritigenin			
